# Genetic Insights into the Rheological Phenotype of Candida albicans Biofilms

**DOI:** 10.21203/rs.3.rs-8555167/v1

**Published:** 2026-01-20

**Authors:** Clémence Abriat, Linqi Huang, Sham Sunder, Joshua S. Bauman, Grant A. Landry, Hunter R. Lischwe Mueller, J. Scott VanEpps, Michael J. Solomon, Anuj Kumar

**Affiliations:** University of Michigan–Ann Arbor

## Abstract

The opportunistic fungal pathogen Candida albicans forms biofilms with viscoelastic properties and rheological response to flow that can significantly impact its biology and life cycle. The rheological phenotypes of fungal biofilms, however, have been neither quantified extensively nor genetically dissected. We have developed methods to quantify the rheology of fungal biofilms, and, here, we analyzed a panel of C. albicans deletion mutants impaired in cell wall structure or extracellular matrix (ECM) production for rheological phenotypes. Increased elastic moduli, indicative of higher viscoelasticity, were evident in strains singly deleted for ALG11, KRE5, and PMR1, with complementation strains showing wild-type phenotypes. The deletion mutants exhibited a smooth biofilm morphology on agar, with reduced hyphae, decreased ECM, and decreased fluconazole resistance. Transcriptional profiling of these strains identified altered expression of genes affecting cell membrane/cell wall biology, translation, protein catabolism, lipid metabolism, and filamentous development. Collectively, the data present C. albicans biofilm rheology as a distinct phenotype affected by ECM production and cell morphology, while identifying genes for the further investigation of fungal biofilm viscoelasticity.

## Introduction

Fungal infections are increasing worldwide, presenting as an important clinical problem with high associated mortality rates ^1-6^. The World Health Organization (WHO) recently published the first list of fungal priority pathogens, and *Candida albicans* was classified within the critical category, as it leads to severe infections and shows resistance to antifungal treatments ^7^. *C. albicans* is the principal opportunistic human fungal pathogen, with multiple virulence modalities including biofilm formation on various surfaces, such as host tissues or medical implants ^8-17^. These complex biofilm structures, composed of cells embedded in an extracellular matrix (ECM), are extremely resistant to currently available antifungal treatments ^18-20^. Biofilm-mediated drug resistance stems from the presence of persister cells, the upregulation of drug-efflux pumps, and ECM sequestration of the antifungal drug, thus restricting its penetration into the biofilm structure ^21-23^.

The *C. albicans* biofilm ECM is composed of proteins, lipids, eDNA, and carbohydrates ^24^. Carbohydrates represent approximately 25% of the ECM and contain a-mannan, b-1,3-glucan, and b-1,6-glucan ^22^. The b-1,3-glucan has been studied for its role in antifungal resistance; disruption of glucan synthesis is associated with a lower resistance to antifungal agents ^25^. ECM polymers contribute to the viscoelasticity exhibited by bacterial biofilms, as assessed through bulk rheology, microrheology, and interfacial rheology ^26-28^. Rheology is the measure of deformation of material, including soft matter, in response to fluid and shear forces. *C. albicans* biofilms are viscoelastic gels with mechanical properties responsive to hydrodynamic conditions of fluid flow, fluctuating pressures, and high shear rates ^29,30^. The mechanics and rheology of biofilms have been studied in several bacterial species, including in *E. coli, S. epidermis, P. aeruginosa, B. subtilis, V. cholerae*, and *K. pneumoniae* ^26,31-35^. Bacterial biofilm rheology structure and properties have been reviewed elsewhere ^30,36-38^. Notably, much less is known regarding the biomechanical properties of fungal biofilms, and relevant research has addressed the topic from industrial, agricultural, and engineering perspectives using fungi other than *C. albicans* ^39,40^. The properties of fungal biofilms are distinct from those of bacterial biofilms, as evidenced by the distinct culturing conditions and methods needed for rheological analysis of *C. albicans* biofilms ^41^. The rheological responses of *C. albicans* biofilms are important to consider, as these responses affect fungal dispersal and, consequently, the outcome of cleansing and bioremediation efforts. We have previously established methods to measure the rheological properties of *C. albicans* biofilms grown on polycarbonate membrane ^41^, and here, we apply these methods to consider inputs that modify *C. albicans* biofilm rheology.

*C. albicans* is capable of dimorphism, existing in yeast-like and filamentous forms. Its yeast form is characterized by the presence of oval planktonic cells that divide by budding, and its filamentous forms encompass pseudohyphal filaments and extended hyphal tubes ^42-44^. During pseudohyphal growth, elongated yeast cells remain physically connected after budding, forming filamentous cellular chains. Hyphae result from incomplete cell division, with the extended hyphal tubes exhibiting parallel-sided cell walls. These different cell morphologies have been associated with different mechanical profiles, as hyphae exhibit less rigidity than yeast-type cells ^45,46^. Moreover, cell wall carbohydrate composition and cell surface roughness also modify the stiffness of yeast cells ^47^. However, it is unclear whether cell shape, cell wall structure, and ECM composition contribute to the viscoelastic properties of *C. albicans* biofilms. Here we identify genes that affect the viscoelasticity of *C. albicans* biofilms cultured on polymer membranes, and we investigate the impact of cell morphology and ECM composition on biofilm mechanical properties by combining rheological, microscopic, biochemical, and transcriptomic analysis.

## Results

### Identifying genes that affect C. albicans biofilm viscoelasticity

We reasoned that cell wall structure and/or ECM composition likely affects *Candida albicans* biofilm viscoelasticity; consequently, we analyzed rheology phenotypes in a panel of mutants deleted for genes encoding proteins that contribute to wild-type fungal cell wall structure or ECM makeup. For these studies, we cultured biofilms from the following deletion mutants: *alg11*Δ/Δ, *chs8*Δ/Δ, *fks1*Δ/*FKS1, kre5*Δ/Δ, *mnn9*Δ/Δ, *mnn22*Δ/Δ, *phr1*Δ/Δ, *pmr1*Δ/Δ, and *van1*Δ/Δ. The *chs8*Δ/Δ, *fks1*Δ/*FKS1, kre5*Δ/Δ, *mnn9*Δ/Δ, *phr1*Δ/Δ, and *pmr1*Δ/Δ mutants were generated by gene replacement in BWP17 ^48^, while *alg11*Δ/Δ, *mnn22*Δ/Δ, and *van1*Δ/Δ were derived from strain SN152 ^49^. Accordingly, we cultured biofilms from both the BWP17 and SN152 wild-type strains for comparison. Strain genotypes are indicated in Supplementary Table 1. The genes *ALG11, MNN9, MNN22*, and *VAN1* encode mannosyltransferases ^50-52^. Mnn9p, Mnn22p, and Van1p are proteins of the Mnn9p family of mannosyltransferases ^50^. *FKS1/GSC1* encodes β-1,3-glucan synthase, and *PHR1* encodes a cell surface glycosidase thought to act on cell wall β-1,3-glucan prior to its linkage to β-1,6-glucan ^53,54^. *CHS8* encodes a class I chitin synthase required for wild-type synthesis of long-chitin microfibrils in the cell wall ^55^. Kre5p is a UDP-glucose:glycoprotein glucosyltransferase ^56^, and Pmr1p is a secretory pathway P-type calcium pump involved in protein glycosylation and cell wall maintenance ^57^.

**Figure 1:**
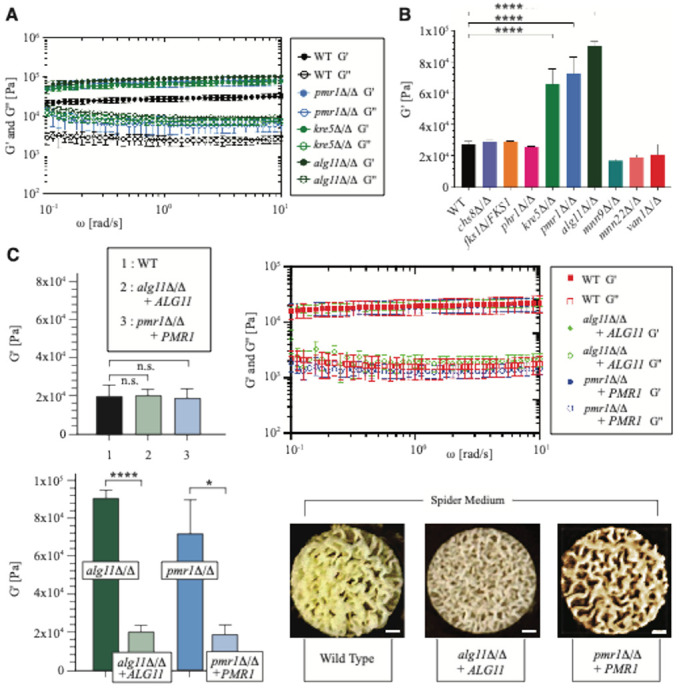
The *alg11*D/D, *kre5*D/D, and *pmr1*D/D biofilms exhibit mechanical properties that differ from wild type. **a** Storage and loss moduli (G¢ and G^2^) were determined as a function of frequency w at g_0_=0.08%. Full datasets are provided in Supplementary File 1. **b** The elastic modulus G¢ at w=1 rad/s and g_0_=0.08% was determined for biofilms from the indicated strains. Error bars indicate standard error of the mean. Significance: ****, *p*-value < 0.0001. The full dataset is provided in Supplementary File 1 and Supplementary File 2. **c** G¢ and G^2^ values are indicated for the complementation strains transformed with a wild-type copy of *ALG11* or *PMR1* (Supplementary File 1). Storage and loss moduli for the wild-type BWP17 and SN152 strains were very similar, and measures of biofilms from both strains are included in the wild-type data presented. Error bars indicate standard error of the mean. Statistical significance was calculated as above. Differences in G¢ and G^2^ values between wild-type and complementation strains were not statistically significant. Storage moduli for biofilms cultured from the complementation strains were statistically significant from values obtained for biofilms from the respective homozygous deletion mutants. Significance: ****, *p*-value < 0.0001; *, *p*-value < 0.05. Biofilm morphologies of the indicated strains cultured on Spider medium with mannitol as a carbon source are shown. Scale bar, 1 mm.

Biofilm mechanical properties were evaluated quantitatively after growth on agar using parallel-plate rheology. All biofilms from the tested *C. albicans* wild-type and mutant strains exhibited solid-like behavior, as their elastic G′ and viscous G3 moduli did not exhibit a frequency dependency (Fig. 1a, Supplementary File 1). The G′ value was also higher than the G″ value, similar to what has been observed for bacterial biofilms ^32^. We did not observe a statistically significant difference in G′ or G″ moduli between the wild-type BWP17 and SN152 strains (Supplementary File 1). The elastic modulus G′ was significantly higher compared to wild type for the following three mutants: *alg11*Δ/Δ, *kre5*Δ/Δ, and *pmr1*Δ/Δ (Fig. 1b, Supplementary File 2). Values for these mutants were more than three times the level of biofilms formed from wild-type strains, suggesting a stiffer material. An amplitude sweep was performed, during which the *alg11*Δ/Δ, *kre5*Δ/Δ, *pmr1*Δ/Δ, and wild-type biofilms were subjected to an increased oscillatory amplitude while maintaining the frequency at 1 rad/s (Supplementary Fig. 1). Biofilms from the mutant strains showed a higher crossover point (the strain at which G′ equals G″), which is the non-linear strain amplitude at which the biofilm transitions between elastic- and viscous-dominated states.

To confirm that the observed rheology phenotypes were due to the intended gene deletions, we analyzed complementation strains with wild-type *ALG11* and *PMR1* introduced into the *alg11*Δ/Δ and *pmr1*Δ/Δ genetic backgrounds, respectively. The *ALG11*- and *PMR1*-complemented strains yielded G′ and G″ moduli with insignificant difference from the wild-type BWP17 and SN152 strains (Fig. 1c). Both complementation strains were significantly different from their respective homozygous deletion parents. Cultured on Spider medium with mannitol as its carbon source, the complementation strains exhibited a biofilm morphology that closely resembled wild-type biofilms (Fig. 1c).

The measurement of bulk rheology of *C. albicans* biofilms and the identification of deletion mutants with distinct biofilm viscoelastic properties establishes biofilm rheological response as a *C. albicans* phenotypic trait for further analysis. Here, we sought to characterize the *alg11*Δ/Δ, *kre5*Δ/Δ, and *pmr1*Δ/Δ mutants as an entry towards understanding factors that influence and/or are associated with fungal biofilm rheology.

### Biofilm structure and cell morphology of mutants with elevated biofilm mechanical properties

**Figure 2:**
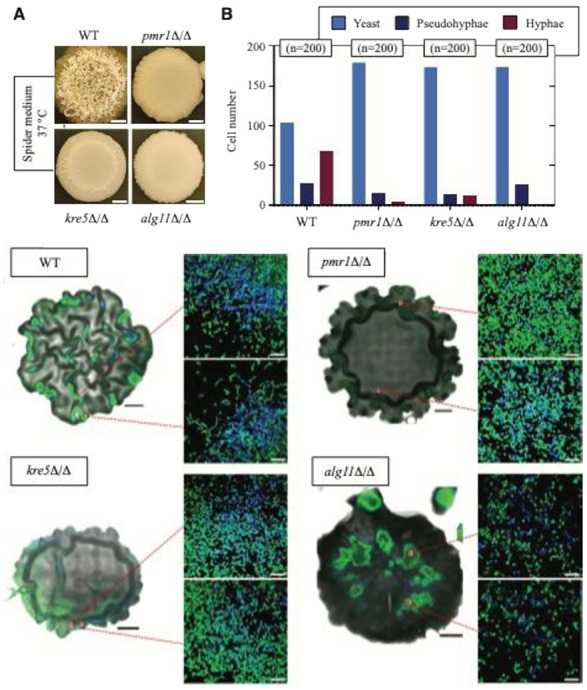
Microscope images of *C. albicans* biofilms. **a**Images of whole colony biofilm morphology are shown for wild-type BWP17, *pmr1*D/D, *kre5*D/D, and *alg11*D/Dstrains grown on solid Spider medium (low-nutrient medium with mannitol). Scale bar, 1 mm. Microscope z-stacked images were acquired for these biofilms, with enlarged insets indicating a field of cells in a single frame as shown. Cells were stained with SYTO 9 (green), and chitin was visualized by staining (blue) with Calcofluor White. Inset scale bar, 30 mm. **b** Cells rom the images were categorized as being yeast-form (cell length:width ratio < 1.5), pseudohyphal (length:width 1.5-2), or hyphal (length:width > 2). Two hundred cells were counted for each indicated strain.

To assess colony biofilm ultrastructure and constituent cell morphology, we imaged *alg11*Δ/Δ, *kre5*Δ/Δ, and *pmr1*Δ/Δ biofilms cultured on agar by confocal microscopy (Fig. 2). For imaging, cells were stained green with SYTO 9, and chitin was stained blue using Calcofluor White. Biofilms were imaged from top to bottom, as we observed previously that *C. albicans* biofilm morphology is heterogeneous in the z-direction, with a smoother morphology at the bottom ^41^. Macroscopic images and large-scale images of whole colony biofilms (top sections) revealed a heterogeneous structure for the wild-type strain, with a wrinkled morphology (Fig. 2a). The *kre5*Δ/Δ and *pmr1*Δ/Δ biofilms are dense and exhibit a more homogenous morphology, with wrinkled regions evident towards the edges of the biofilm. The biofilm formed by *alg11*Δ/Δ is very smooth, and the structure started to dissolve when incubated in the dye solution. Higher magnification confocal images indicate decreased numbers of hyphae and pseudohyphal cells in the biofilms formed by *alg11*Δ/Δ, *kre5*Δ/Δ, and *pmr1*Δ/Δ, as quantified in Fig. 2b.

### ECM and carbohydrate analysis of alg11Δ/Δ, kre5Δ/Δ, and pmr1Δ/Δ biofilms

**Figure 3:**
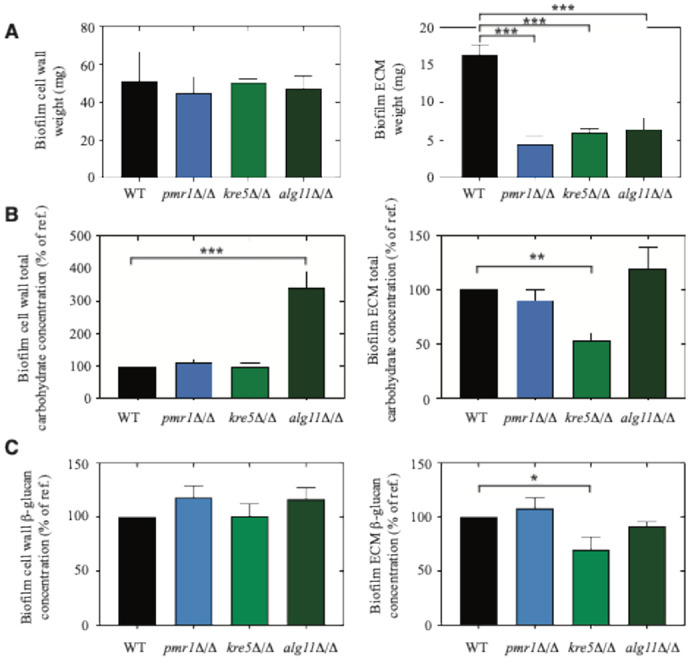
Analysis of biofilm composition. **a** The cell wall and ECM dry mass (mg) of wild-type, *pmr1*D/D, *kre5*D/D, and *alg11*D/Dbiofilms are shown. Error bars indicate standard error of the mean. **b**Carbohydrate concentrations (mg/L) of biofilm cell wall and ECM fractions are shown. Error bars were calculated as above, and the full dataset is provided in Supplementary File 3. **c** Total b-glucan levels were determined for the cell wall and ECM fractions extracted from biofilms cultured as described. b-glucan concentrations obtained for wild-type BWP17 were set as reference levels, and data are presented as percentages of the reference level. Error bars were generated as described above, and File S4 contains individual data for this analysis. Significance: *, *p*-value < 0.05; **, *p*-value < 0.01; ***, *p*-value < 0.001.

We analyzed *alg11*Δ/Δ, *kre5*Δ/Δ, and *pmr1*Δ/Δ biofilms grown on agar for cell wall weight, ECM weight, and carbohydrate concentration. We did not observe a statistically significant change in cell wall weight for any of the mutants relative to wild type. ECM dry weight, however, was significantly decreased in biofilms grown from *alg11*Δ/Δ, *kre5*Δ/Δ, and *pmr1*Δ/Δ relative to wild type (Fig. 3a). The total carbohydrate levels of both the biofilm matrix and cell wall were quantified using the modified phenol-sulfuric acid method described by Ogura *et al.* ^58^. Total carbohydrate concentration was significantly elevated relative to wild type in the cell wall fraction of the collected *alg11*Δ/Δ biofilm, while carbohydrate concentration was similar to wild type in the cell wall component of *kre5*Δ/Δ and *pmr1*Δ/Δ biofilms (Fig. 3b, Supplementary File 3). In N-linked glycosylation in the rough endoplasmic reticulum, Alg11p uses the nucleotide sugar donor substrates UDP-Glc*N*Ac and GDP-Man to synthesize an intermediate of the lipid dolichol (Dol-PP- Glc*N*Ac_2_Man_5_) towards generation of the Glc*N*Ac_2_Man_8_ structure ^59^. It has been previously observed that cells with defects in *N*-linked mannan biosynthesis exhibit altered cell wall composition, with elevated glucan content ^60,61^. The UGGT1 glucosyltransferase Kre5p works at a later step in the N-linked glycosylation pathway to reglucosylate misfolded protein. Kre5p activity may not significantly impact total cell wall carbohydrate content, while still contributing to glycoprotein synthesis and ER-mediated quality control ^62^. We analyzed total carbohydrate concentration in the ECM from biofilms formed by *alg11*Δ/Δ, *kre5*Δ/Δ, and *pmr1*Δ/Δ relative to wild type, and ECM total carbohydrate levels were decreased in *kre5*Δ/Δ biofilms (Fig. 3b, Supplementary File 3). We measured β-glucan levels, encompassing β-1,3-glucan and β-1,6-glucan, in the cell wall and ECM of biofilms for the respective mutants and wild-type strains grown as described (Fig. 3c, Supplementary File 4). Levels of β-glucan in the cell wall were not significantly different from wild-type in biofilms for *alg11*Δ/Δ, *kre5*Δ/Δ, and *pmr1*Δ/Δ. ECM β-glucan levels were significantly decreased in the *kre5*Δ/Δ biofilm relative to wild type (Fig. 3c), consistent with decreased total carbohydrate levels observed in the ECM of *kre5*Δ/Δ biofilms.

### Resistance to fluconazole is decreased in alg11Δ/Δ, kre5Δ/Δ, and pmr1Δ/Δ biofilms

**Figure 4:**
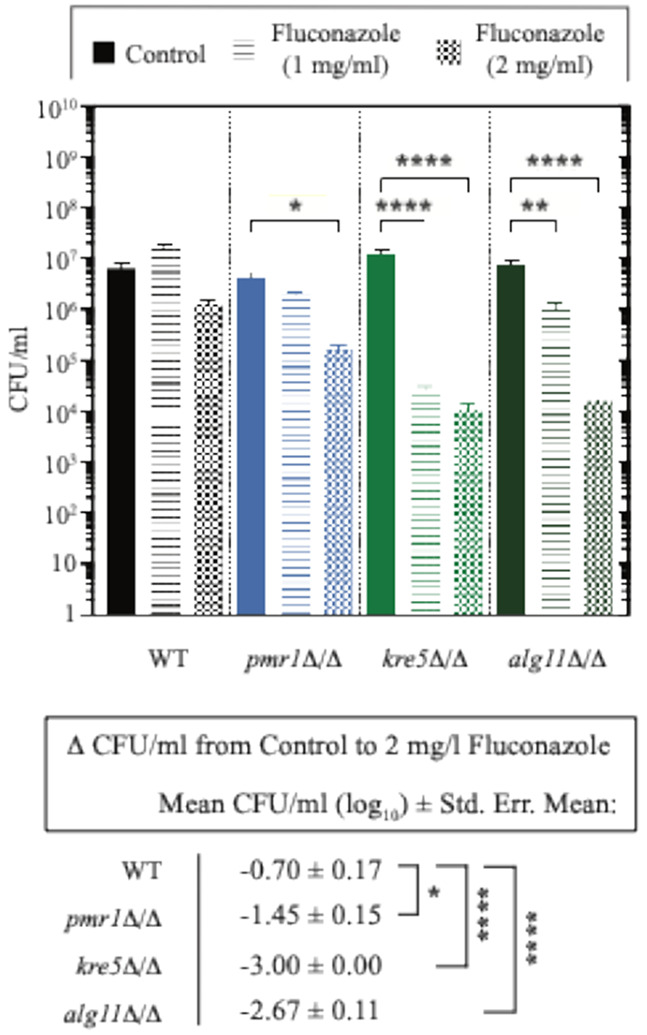
The *alg11*D/D, *kre5*D/D, and *pmr1*D/Dbiofilms show decreased resistance to fluconazole relative to wild type. *C. albicans* cell viability was assessed by counting colony-forming units (CFU)/ml from biofilms cultured as described after fluconazole treatment at 1 mg/ml or 2 mg/ml. Error bars indicate standard error of the mean. Significance: *, *p*-value < 0.05; **, *p*-value < 0.01; ***, *p*-value < 0.001; ****, *p*-value < 0.0001. Full datasets are provided in Supplementary File 5.

*C. albicans* biofilms are resistant to antifungal agents, such as fluconazole ^22,63,64^. To consider if biofilms with enhanced mechanical properties also exhibit an altered response to antifungal agents, we grew wild-type and *alg11*Δ/Δ, *kre5*Δ/Δ, and *pmr1*Δ/Δ biofilms on agar and subjected the samples to two 24-hour treatments with fluconazole at concentrations of 1 mg/ml and 2 mg/ml. Treated biofilms were sonicated to release cells from the ECM, and cells were counted for the determination of viable colony-forming units (CFUs) (Fig. 4, Supplementary File 5). Wild-type *C. albicans* did not exhibit a significant reduction in CFU/ml at either fluconazole concentration. Cells in the *alg11*Δ/Δ and *kre5*Δ/Δ biofilms showed a significant reduction in CFU/ml upon treatment with 1 mg/ml of fluconazole. Cells in the *alg11*Δ/Δ, *kre5*Δ/Δ, and *pmr1*Δ/Δ biofilms exhibited a statistically significant reduction in CFU/ml count with treatment of 2 mg/ml fluconazole (Fig. 4). The decrease in CFU/ml count from treatment with no fluconazole to treatment with 2 mg/ml fluconazole is significantly greater in *alg11*Δ/Δ, *kre5*Δ/Δ, and *pmr1*Δ/Δ than the corresponding change in wild type. These data indicate that biofilms formed by the tested deletion mutant strains show decreased resistance to fluconazole relative to biofilms formed by a wild-type strain similarly cultured on agar. From the preceding studies, these mutant biofilms also exhibit enhanced elasticity, decreased hyphal content, and lower ECM dry mass.

### Genome-wide transcriptional profiling of cells in biofilms with altered viscoelasticity

We complemented the above characterizations of cell and ECM properties in the *alg11*Δ/Δ, *kre5*Δ/Δ, and *pmr1*Δ/Δ biofilms by generating genome-wide profiles of differentially abundant RNA transcripts in cells from these biofilms. For these studies, we grew wild-type and deletion mutant biofilms on agar as before and extracted cells from the biofilms. Purified RNA was subsequently analyzed by high-throughput sequencing as a molecular readout of differentially active processes in the respective cells.

**Figure 5:**
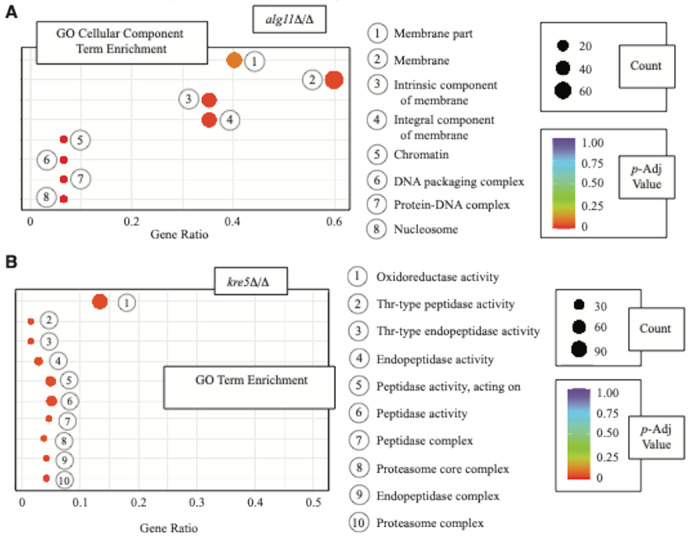
Gene Ontology term enrichment in differentially abundant transcripts for *alg11*D/Dcells and *kre5*D/Dcells extracted from cultured biofilms relative to wild type. **a** Genes with differential transcript levels in *alg11*D/Drelative to wild type were analyzed for annotated Gene Ontology terms enriched over background. Several GO Cellular Component terms were enriched in the set of genes differentially expressed in *alg11*D/D; these terms are shown as a dot plot. GO terms are numbered and represented by circles, as indicated in the accompanying key to the right. The X-axis identifies the ratio of genes in the differential transcript dataset annotated with the indicated GO term as compared against genes annotated with the identical term in the genome as a whole. The size of the dot is proportional to the number of genes with the given GO term, and the Benjamini and Hochberg adjusted *p*-value of the enrichment is color-coded as shown. The set of transcripts differentially abundant in *alg11*D/Dis enriched for genes encoding membrane-localized proteins. Full datasets with unadjusted *p*-values are provided in Supplementary Files 6 and 7 for analyzed GO Biological Process and KEGG terms, respectively. **b** GO Biological Process terms enriched in differentially abundant transcripts in *kre5*D/Dare presented as above. Genes annotated with enriched GO Biological Process terms (Supplementary Fig. 3a) and KEGG terms (Supplementary Fig. 3b) are provided. Full datasets with unadjusted *p*-values are presented in Supplementary Files 8 and 9, respectively.

The *alg11*Δ/Δ cells, relative to wild-type, exhibit an altered transcriptional program. The set of differentially abundant transcripts in *alg11*Δ/Δ is enriched in genes that encode transmembrane or membrane-associated proteins by analysis of Gene Ontology (GO) Cellular Component terms (Fig. 5a). This is consistent with *ALG11* function in endoplasmic reticulum-mediated glycosylation of membrane proteins. Transcripts differentially abundant in *alg11*Δ/Δ cells relative to wild type identify genes annotated with GO Biological Process terms of lipid catabolism, transmembrane transport, and amino acid metabolism/biosynthesis (Supplementary Fig. 2a). Analysis of this gene set using annotations from the Kyoto Encyclopedia of Genes and Genomes (KEGG) highlights a subset of genes enriched in ubiquinone biosynthesis, proteasomal function, porphyrin metabolism, and the biosynthesis of hydrophobic branched-chain amino acids (Supplementary Fig. 2b). A full listing of differentially abundant genes in *alg11*Δ/Δ and annotated GO Biological Process and KEGG terms for these genes is provided in Supplementary File 6 and Supplementary File 7, respectively.

Cells extracted from *kre5*Δ/Δ biofilms exhibit a transcriptional profile showing differential expression of genes associated with proteolysis and protein catabolism. Differentially abundant transcripts in *kre5*Δ/Δ cells are enriched for genes with GO Cell Component terms associated with the proteasome and GO Molecular Function terms indicating peptidase activity (Fig. 5b). Transcripts of genes involved in protein catabolism and aminoacylation are decreased in abundance relative to wild type (Supplementary Fig. 3a, Supplementary File 8). A partially overlapping set of genes involved in proteolysis predominantly shows decreased transcript levels in *kre5*Δ/Δ. Genes annotated as functioning in secretion and exocytosis and membrane lipid metabolism show increased transcript levels in *kre5*Δ/Δ relative to wild type. Analysis of annotated KEGG terms associated with genes differentially expressed in *kre5*Δ/Δ shows an enrichment for processes of amino acid metabolism and inositol phosphate metabolism (Supplementary Fig. 3b, Supplementary File 9).

**Figure 6:**
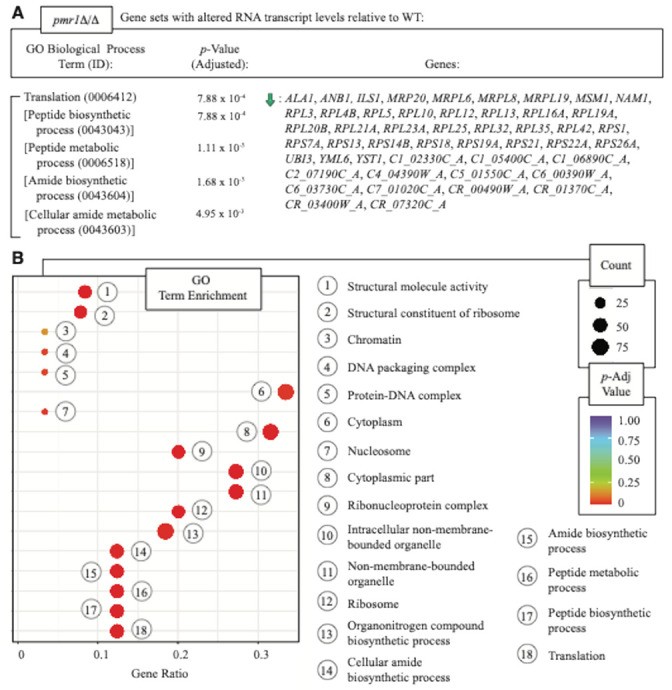
GO terms enriched in the set of differentially abundant transcripts in *pmr1*D/Drelative to wild type. **a** Genes with differential transcript levels in *pmr1*D/D were analyzed for annotated Gene Ontology Biological Process terms enriched over background. IDs for the enriched GO terms are shown with annotated genes, and highly related GO terms are bracketed together. Adjusted *p*-values indicating significance are shown, and genes showing decreased transcript abundance relative to wild type are shown with a green arrow pointing downward. The full dataset with unadjusted *p*-values is shown in Supplementary File 10. **b** GO terms enriched above background for the set of genes with differential transcript abundance are shown in the dot plot. The set of differentially expressed transcripts in *pmr1*D/Dis enriched in proteins involved in translation and peptide biosynthesis. The transcript set is enriched in proteins that localize to ribosomes and nucleosomes. KEGG terms enriched in the dataset are provided in Supplementary File 11.

The transcriptional program in *pmr1*Δ/Δ shows a statistically significant decrease in RNA levels for genes involved in processes related to protein translation (Fig. 6a). The set of transcripts differentially abundant in *pmr1*Δ/Δ is enriched for genes with GO Cellular Component terms identifying the cytoplasm, ribosome, and nucleosome (Fig. 6b). Genes associated with lipid metabolism show decreased and increased transcript levels relative to wild type in cells extracted from *pmr1*Δ/Δ (Supplementary File 10, Supplementary File 11).

**Figure 7:**
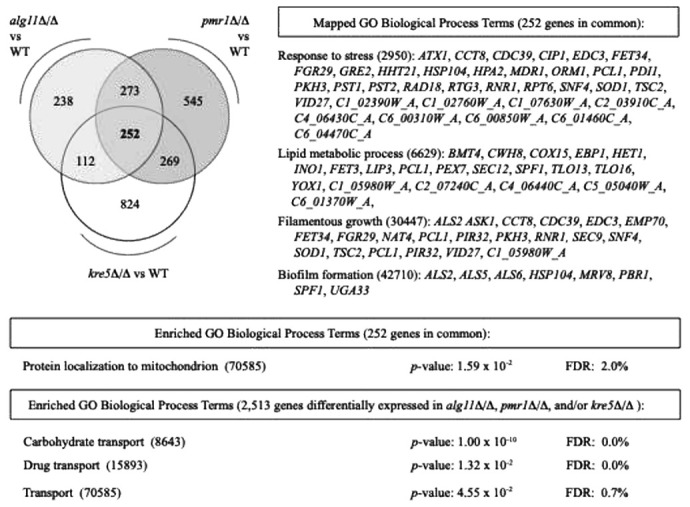
Gene Ontology Biological Process terms associated with gene transcripts differentially abundant in cells from *alg11*D/D, *kre5*D/D, and/or *pmr1*D/Dbiofilms. The Venn diagram indicates transcripts differentially abundant relative to wild type in the respective data sets. In total, 252 genes were differentially expressed in the intersection set of transcriptional profiling data from each of *alg11*D/D, *kre5*D/D, and *pmr1*D/D. Genes in this set that map to the following GO Biological Process terms are shown: Response to stress (ID:2950), Lipid metabolic process (ID:6629), Filamentous growth (ID:30447), Biofilm formation (ID:42710). The full set of annotations is listed in Supplementary File 12. This set of 252 genes is enriched for genes that encode proteins localizing to the mitochondrion. False discovery rate (FDR) is shown with adjusted *p*-value. The union set of transcripts differentially abundant in *alg11*D/D, *kre5*D/D, and *pmr1*D/Dencompasses 2,513 genes; GO Biological Process terms associated with carbohydrate and drug transport are enriched in this data set, with adjusted *p*-values and FDR indicated. The full enrichment data set with unadjusted *p*-values is provided in Supplementary File 13.

The transcriptional profiles for cells in *alg11*Δ/Δ, *kre5*Δ/Δ, and *pmr1*Δ/Δ biofilms are distinct, but the intersection set of differentially abundant transcripts in each of the profiles (252 genes in total) is enriched for genes encoding mitochondrial proteins (Fig. 7, Supplementary File 12). This gene set encompasses *AFG3, BCS1, COX17, CYT2, MAM33, NAM2, OCT1, OXA1, PET100, QCR7, TIM9, TIM44, TIM50, TOM20*, and *TOM40*; these genes are predominantly repressed in the mutants relative to wild type. Mitochondrial processes, including glycolysis and respiration, are required for wild-type biofilm growth ^65^, and many of these genes, including *COX17, OXA1, TIM9, TIM44, TIM50*, and *TOM40*, are repressed upon growth on Spider medium ^66^. The set of genes with differentially abundant transcripts in *alg11*Δ/Δ, *kre5*Δ/Δ, and *pmr1*Δ/Δ is annotated with additional GO Biological Process terms that may be relevant to *C. albicans* biofilm properties, including response to stress (GO: 2950), lipid metabolic process (GO: 6629), filamentous growth (GO: 30447), and biofilm formation (GO: 42710) (Fig. 7, Supplementary File 12). The union set of genes differentially expressed in cells of either *alg11*Δ/Δ, *kre5*Δ/Δ, or *pmr1*Δ/Δ biofilms (2,513 unique genes) is enriched in genes annotated with GO Biological Process terms indicating membrane transport (Fig. 7, Supplementary File 13).

## Discussion

In this study, we analyzed a selected set of mutants deleted for genes contributing to cell wall structure or ECM composition as a means of identifying genes that affect *C. albicans* biofilm rheological properties. Biofilms formed on agar from *alg11*Δ/Δ, *kre5*Δ/Δ, and *pmr1*Δ/Δ strains exhibited a higher elastic modulus than wild type. These biofilms were densely packed and more homogenous in structure than wild type, and cells in the respective biofilms showed decreased filamentous development. Biofilm ECM production was decreased in each of these strains. Total carbohydrate content was elevated in the extracted *alg11*Δ/Δ cell wall fraction, and total carbohydrate and β-glucan levels were decreased in the ECM from *kre5*Δ/Δ biofilms. Treatment of the *alg11*Δ/Δ, *kre5*Δ/Δ, and *pmr1*Δ/Δ biofilms with high concentrations of fluconazole indicated decreased resistance to the antifungal agent relative to wild type. Transcript levels for genes associated with membrane functions, protein catabolism, and translation were altered relative to wild type in cells extracted from the *alg11*Δ/Δ, *kre5*Δ/Δ, and *pmr1*Δ/Δ biofilms. Altered transcript levels were observed for genes involved in filamentous development and biofilm formation in these cells.

Collectively, the data identify fungal biofilm mechanical properties as an informative phenotype in considering biofilm development. The genetic perturbations resulted in increased mechanical properties, and the mutants exhibited decreased hyphal development and ECM production. This increased elastic modulus may reflect, in part, the increase in yeast-form cells observed in biofilms of *alg11*Δ/Δ, *kre5*Δ/Δ, and *pmr1*Δ/Δ, since yeast-form cells exhibit greater rigidity than hyphae ^45,46^. Biofilm viscoelasticity may also be affected by cell density. In previous studies, we found that environmental stressors, such as osmotic stress, affect local cell density in *S. epidermis* biofilms ^67^, and the rheological properties of these biofilms are affected by salt concentration in the growth media ^26^. A direct link, however, between cell density and biofilm viscoelasticity has not been established. It is likely that the degree of filamentous growth and levels of ECM production contributed to the altered viscoelasticity of the biofilm, although those factors are insufficient to explain the phenotype. It is unlikely that defects in hyphal development exclusively drive elevated biofilm mechanical properties, as *chs8*Δ/Δ biofilms, for example, show decreased hyphal growth with an elastic modulus comparable to wild type. ECM production has not been characterized as widely as hyphal growth phenotypes, but *phr1*Δ/Δ is affected in the accumulation of mature matrix biomass ^21^, and its biofilm did not exhibit an elevated elastic modulus in this analysis. Total carbohydrate content and β-glucan levels varied between *alg11*Δ/Δ, *kre5*Δ/Δ, and *pmr1*Δ/Δ biofilms, despite the consistently elevated biofilm mechanical properties of the mutants, with a change from wild-type levels evident in the *kre5*Δ/Δ biofilm ECM. The data are consistent with multiple factors, including cell morphology, cell density, ECM production, carbohydrate content, and β-glucan levels contributing to biofilm mechanical properties, with no single factor in isolation sufficient to drive the phenotype.

It is important to note that in order to grow sufficient material for rheological analysis, the *C. albicans* biofilms in these studies were cultured for an extended period of time. We also induced biofilm formation using solid nutrient-limited medium with mannitol (Spider medium). The extended growth period and use of Spider medium, as opposed to growth in yeast extract-peptone-dextrose (YPD) or RPMI 1640 medium, may affect these results relative to other studies ^68^. The biofilms for this work were grown *in vitro* using laboratory strains of *C. albicans*, and we have not attempted to replicate growth conditions in a host environment.

Cells in the *alg11*Δ/Δ, *kre5*Δ/Δ, and *pmr1*Δ/Δ biofilms exhibited distinct transcriptional profiles, constituting a molecular readout of activity within the cells. The set of transcripts with altered abundance highlight genes important in determining cell morphology, cell wall structure, and ECM composition. These differentially expressed genes, particularly transcripts differentially abundant in all three mutants, provide data for the further identification of factors affecting *C. albicans* biofilm mechanical properties. Genes involved in filamentous development, cell wall architecture, and ECM production are likely candidates to affect fungal biofilm viscoelasticity.

The decreased fluconazole resistance of *alg11*Δ/Δ, *kre5*Δ/Δ, and *pmr1*Δ/Δ biofilms cultured on solid medium is notable. The diminished ECM mass in these biofilms may effectively trap less fluconazole, affording the antifungal agent greater access to the cells and decreased cell viability ^69^. Fluconazole affects the ergosterol biosynthesis pathway in *C. albicans* ^70^, and genes associated with lipid metabolism were differentially expressed in the mutant biofilms. Transcripts encoding membrane proteins were differentially abundant in *alg11*Δ/Δ cells, and genes involved in drug transport were enriched in the union set of differentially expressed transcripts in *alg11*Δ/Δ, *kre5*Δ/Δ, and *pmr1*Δ/Δ.

In sum, the data present fungal biofilm rheological response as a phenotypic output affected by numerous inputs. The viscoelasticity phenotypes of *C. albicans* biofilms are at least partially distinct from cell morphology phenotypes, cell wall structure phenotypes, and biofilm mass measurements. To determine more fully the genetic factors that influence biofilm mechanical properties, a larger set of *C. albicans* deletion mutants will need to be screened. For this purpose and the required scale of analysis, the development of methods for high-throughput fungal biofilm microrheology becomes an important objective.

## Methods

### Growth conditions

*C. albicans* strains were taken from stock frozen at −80°C and grown in culture overnight by shaking in YPD supplemented with uridine at 37°C. As described previously, biofilms were grown from overnight cultures on polycarbonate membranes (Cytiva Whatman, Marlborough, MA, Nucleopore 0.8 μm pore size) placed on solid Spider medium supplemented with uridine for seven days at 37°C ^41,71^.

### Strains and strain construction

Strains used in this study are listed in Supplementary Table 1. Deletion mutants were constructed by fusion PCR-based methods ^49^. In brief, PCR was used to amplify 350–500 bp of chromosomal DNA flanking the gene targeted for deletion. PCR was also used to amplify the *C. dubliniensis ARG4* or *HIS1* gene as an auxotrophic marker using primers containing 20 nt of 5’-sequence complementary to the amplified sequence flanking the target gene. The three overlapping products were used as template for PCR amplification to generate a final product with the auxotrophic marker and gene-flanking regions. Standard DNA transformation protocols were used to introduce the PCR product into *C. albicans*. Integration of the auxotrophic marker by homologous recombination was selected by growth on appropriate medium. *HIS1* and *ARG4* were used to replace both copies of the targeted gene. One copy of *FKS1* was replaced with *HIS1*, as the gene is essential. The heterozygous mutant *fks1*Δ /*FKS1* was used for the studies here. At least two independent transformants were selected for each deletion mutant, and similar growth properties were confirmed for each transformant strain.

To construct the *PMR1*^+^ complementation strain, the wild-type *PMR1* gene with 500 bp of upstream sequence and 200 bp of downstream sequence was cloned into a pUC19-based vector carrying the pFA-*SAT1* cassette. The resulting plasmid was linearized for integration at the *PMR1* locus. Integrants were selected on media supplemented with uridine (80 μg/ml) and nourseothricin (200 μg/ml). Integration of wild-type *PMR1* was confirmed by PCR. The same approach was used to clone wild-type *KRE5*, and the resulting linearized vector was prepared for introduction into *kre5*Δ/Δ by DNA transformation; however, we were unable to recover any transformants with the wild-type *KRE5* gene.

### Rheology

*C. albicans* biofilms were grown as described previously to a thickness between 250 μm and 1 mm for rheological measurements ^41^. Seventy-five microliters of a culture grown overnight was placed on a polycarbonate membrane inside a washer to constrict biofilm growth on a plate with Spider medium. The plate was incubated at 37°C. After seven days, the washer was carefully removed, and the polycarbonate membrane with biofilm was transferred to the TA Instrument rheometer Peltier plate set at 20°C. The biofilm was cut with an 8 mm biopsy punch (Integra Miltex, Princeton, NJ). An 8 mm flat parallel plate geometry with self-adhesive sandpaper was lowered until the normal force reached 0.1N, as determined previously ^41^. The oscillatory rheological measurements were performed as follows: a frequency sweep from 0.1 to 10 rad.s^− 1^ was done at a fixed deformation of γ_0_ = 0.08%, giving the linear elastic *G’* and viscous *G”* moduli. Subsequently, a strain sweep was performed from 0.1% to 200% at ω = 1 rad.s^− 1^ to yield non-linear rheology. All tests were carried out with biological triplicates, and the mean ± standard error of the mean was reported. Significance was determined using two-way ANOVA with Dunnett’s multiple comparison test.

### Confocal microscopy

After growth for one week at 37°C on solid Spider medium, biofilms were transferred to a two-well chamber. The chamber was inverted and incubated for 30 minutes with 20 μM SYTO 9 (Invitrogen Life Technologies, Waltham, MA) ^72^, staining all yeast cells, and 1μl Calcofluor White (Sigma-Aldrich, St. Louis, MO), a non-specific dye staining the chitin in *C. albicans* membrane. The biofilms were imaged at the coverslip using a Nikon A1RSi confocal microscope, with a 10x objective (numerical aperture 0.3, 2.49 μm/px) for large scale images and a 60x objective (numerical aperture 1.4, 0.41 μm/px) for localized imaging.

### Carbohydrate measurements

Cultured biofilms were collected and diluted in 1 ml of deionized water. Total carbohydrate content was quantified using the phenol-sulfuric acid method, adapted for a microplate format ^58^. Diluted biofilms were subjected to sonication using an ultrasonic probe (Cole-Parmer, Vernon Hills, IL) with 10 seconds on/10 seconds off pulses at 30% amplitude for 90 seconds. Samples were centrifuged at 4,000 rpm for 10 minutes to separate the ECM from the cell wall fraction. The pellet was resuspended in 4 ml of deionized water. Sulfuric acid (150 μl) was added to 30 μl of either the cell wall or ECM fractions in a 96-well microplate. The plate was heated for 15 minutes at 90°C, and 30 μl of 5% phenol was added to the plate. Samples were allowed to rest for at least 60 minutes. Sample absorbance was measured at 490 nm. A calibration curve of glucose was used to quantify carbohydrate concentration. For measurements of β-glucan, the cell wall fraction was separated from the ECM as for measurement of total carbohydrates. Samples were lyophilized (LabConco, Kansas City, MO) for 24 hours. Dry samples were weighed, and total β-glucan was quantified for the cell wall and ECM using the enzymatic yeast β-glucan assay kit (Megazyme, Wicklow, Ireland). Significance was determined using ordinary two-way ANOVA with Dunnett’s multiple comparison test or Welch’s correction.

### Fluconazole susceptibility assays

Biofilms were grown from 75 μl of an overnight culture on a 19 mm polycarbonate membrane on solid Spider medium supplemented with uridine ^71^. After one week of growth at 37°C, the membrane was carefully transferred to a 24-well plate ^41^. The biofilms were subjected to two 24-hour fluconazole (Sigma-Aldrich, St. Louis, MO) treatments at 37°C ^73^, with concentrations of 1 mg/ml or 2 mg/ml in Spider media supplemented with uridine. After the two treatments, biofilms were subjected to sonication as described with 10-second on/off pulses at 30% amplitude for 90 seconds. Sonicated biofilms were diluted and plated on YPD agar to assess viability. All tests were carried out using at least two biological replicates with three technical replicates. Mean colony forming units (CFU)/ml ± standard error of the mean was reported. Significance was determined using the Kruskal-Wallis test with uncorrected Dunn’s test for multiple comparisons.

### Transcriptomic analysis

Biofilms were grown and cell pellets were prepared as described. RNA was extracted from each sample using the RiboPure RNA purification kit (Thermo Fisher Scientific, Waltham, MA) according to suggested protocols and published methods ^74-76^. Approximately 10^7^ cells were lysed by agitation with cold Zirconia beads in a buffer containing SDS (1% final volume) and phenol:chloroform:isoamyl alcohol. The RNA was purified by ethanol washes and filtration using cartridges in the RiboPure kit. Prior to sequencing, approximately 3 μg of each RNA sample was treated with DNase I. The reaction was terminated with EDTA and incubation at 65°C. The samples were subsequently precipitated with ethanol and washed with 70% cold ethanol.

RNA quality control and sequencing was performed by Novogene (Sacramento, CA). Messenger RNA was purified from total RNA using poly-T oligonucleotide-attached magnetic beads. cDNA was generated using random hexamer primers for first-strand synthesis, and dUTP-priming for second-strand synthesis. The resulting cDNA library was quantified using fluorometry and real-time PCR. The size distribution of the synthesized cDNA was assessed using a bioanalyzer. Quantified libraries were pooled and sequenced on Illumina platforms, according to effective library concentration and data amount.

Sequences in fastq format were processed through custom Perl scripts (Novagene, Sacramento, CA) to obtain reads free of adapter sequences, ambiguous nucleotide determinations, and low-quality sequence. Quality control data and GC -content was determined. Sequences were mapped to *C. albicans* genes using Hisat2 v2.0.5 ^77^, and the number of reads mapped to a given gene was determined using featureCounts v1.5.0-p3 ^78^. Expression levels were estimated by determining the expected number of fragments per kilobase of transcript sequence per millions base pairs sequenced (FPKM) for each gene. Read counts for each library were adjusted by the edgeR program package through one scaling-normalized factor. Differential expression analysis of three biological replicates for each mutant strain versus wild type was performed using the DESeq2 R package (v 1.20.0), providing statistical routines for determining differential expression in digital gene expression data using a model based on the negative binomial distribution. Resulting *p*-values were adjusted according to the approach of Benjamini and Hochberg for controlling false discovery rate. Genes with an adjusted *p*-value of less than or equal to 0.05 as determined by DESeq2 were considered to be differentially expressed. Gene Ontology (GO) ^79^ enrichment analysis of differentially expressed genes was implemented using the clusterProfiler package in R, correcting for gene length bias. GO terms with corrected *p*-values of less than 0.05 were identified as being significantly enriched in the data sets of differentially expressed genes. The identical approach was used to determine enrichment of annotated terms using data in the Kyoto Encyclopedia of Genes and Genomes (KEGG) database ^80^.

## Supplementary Material

This is a list of supplementary files associated with this preprint. Click to download.

- SuppTablesFigs.pdf
- SupplementaryFileLegends.docx
- FileS1draft2.xlsx
- FileS2.xlsx
- FileS3.xlsx
- FileS4.xlsx
- FileS5.xlsx
- FileS6.xlsx
- FileS7.xls
- FileS8.xlsx
- FileS9.xlsx
- FileS10.xlsx
- FileS11.xlsx
- FileS12.xlsx
- FileS13.xlsx

## Data Availability

Strains and reagents are available upon request. Data supporting the findings of this study are available within this paper and its Supplementary Information. Transcriptional profiling data were deposited into the Gene Expression Omnibus database under accession number GSE276413.
